# Retrospective analysis of risk factors for low 1-minute Apgar scores in term neonates

**DOI:** 10.1590/1414-431X20199093

**Published:** 2019-11-28

**Authors:** Congmei Yang, Xia Chen, Shuiling Zu, Fangjie He

**Affiliations:** 1 Department of Obstetrics and Gynecology, The Second Affiliated Hospital of Fujian Medical University, Quanzhou, Fujian, China; 2 Department of Obstetrics and Gynecology, Nanfang Hospital, Southern Medical University, Guangzhou, Guangdong, China; 3 Nursing Department, The Third Affiliated People's Hospital of Fujian University of Traditional Chinese Medicine, Fuzhou, Fujian, China

**Keywords:** 1-min Apgar scores, Term neonates, Second stage of labor, Vaginal delivery, Meconium-stained amniotic fluid

## Abstract

The current study was designed to investigate the perinatal risk factors for low 1-min Apgar scores in term neonates. We retrospectively analyzed the maternal and neonatal clinical data of 10,550 infants who were born through vaginal delivery from 37 weeks 0 days to 41 weeks 6 days of single gestation from January 2013 to July 2018. Because the 1-min Apgar score reflects neonatal status at birth, we analyzed the risk factors for low (score <7) 1-min Apgar scores through logistic regression. Among these 10,550 neonates, 339 (3.2%) had low (score <7) 1-min Apgar scores. Among them, 321 (94.7%) were admitted to the neonatology department for further observation or treatment. Multivariate analysis revealed that educational background, body mass index, gestational age, pathological obstetrics, longer duration of the second stage of labor, forceps delivery or vacuum extraction, neonatal weight, neonatal sex, and meconium-stained amniotic fluid were independent risk factors for 1-min Apgar scores <7. Neonates who had low 1-min Apgar scores were more frequently admitted to the neonatology department for further observation or treatment. Early detection of risk factors and timely intervention to address these factors may improve neonatal outcomes at birth and reduce the rate of admission to the neonatology department.

## Introduction

Dr. Virginia Apgar created the Apgar score in 1952. Since then, it has been used worldwide for the rapid and standardized assessment of neonates after delivery and for the assessment of the need for prompt intervention to establish breathing at 1 min of age (1). The Apgar score comprises the following five clinical signs: heart rate, respiratory effort, muscle tone, reflex irritability, and color. Each of these components is assessed and assigned a value of 0, 1, or 2, and the sum of these components is the final score. The score is reported at 1 and 5 min after birth for all neonates, and at 5-min intervals thereafter until 20 min for neonates with a score less than 7. Even after more than half a century since the Apgar score was integrated into routine clinical practice, it remains a standardized, effective, and convenient tool for neonatal assessment (2).

The 1-min Apgar score reflects the neonatal status at birth and response to resuscitation; however, it has not been shown to be associated with long-term clinical outcomes (3). Population studies have uniformly reassured us that most neonates with low Apgar scores will not develop cerebral palsy. However, a low 5-min Apgar score clearly confers an increased relative risk of cerebral palsy. This increase in the relative risk of cerebral palsy is reported to be as high as 20- to 100-fold greater than that in infants with a 5-min Apgar score of 7 to 10 (4). However, to the best of our knowledge, there is no study assessing the association between prenatal risk factors and low 1-min Apgar scores at birth in neonates.

The aim of this study was to identify the perinatal risk factors for 1-min Apgar scores <7 in singleton neonates born vaginally from 37 weeks 0 days to 41 weeks 6 days of gestation. Early detection of risk factors and timely intervention to address these risk factors may improve neonatal outcomes at birth and reduce the rate of admission to the neonatology department.

## Material and Methods

### Patients

The current retrospective study was approved by the Institutional Review Board at the Second Affiliated Hospital of Fujian Medical University (ethical approval number: 2019-217). Since this study used previously collected human data, the requirement for obtaining informed consent was waived. This study focused on the care of newborns, especially neonates with low (score <7) 1-min Apgar scores. The medical records were collected from mothers and their neonates born through vaginal delivery anytime from 37 weeks 0 days to 41 weeks 6 days of single gestation at the Second Affiliated Hospital of Fujian Medical University from January 2013 to July 2018. The clinical data for 10,550 neonates and their mothers included in this study were obtained from the hospital's digital system. Basic demographic data, such as mothers’ age and educational background, were exported automatically, and two doctors collected the other detailed clinical information from scanned digital records and ensured the correctness of data with Epidata (Odense, Denmark). Some mothers were in the first or second stage of labor when they were admitted to the hospital. They were asked about the time of onset of uterine contractions, the degree and interval of pain, and symptoms of bleeding and leakage of amniotic fluid from the vagina. All women seen in the Department of Obstetrics underwent partography, and women with abnormal results received intervention that included oxytocin to promote uterine contractions, head rotation, forceps delivery, and vacuum extraction. Neonates were included in the study if the following criteria were met: 1) neonate mothers from 37 weeks 0 days to 41 weeks 6 days of single gestation who underwent vaginal delivery in the supine position; 2) neonate mothers with pregnancy complicated by pathological obstetrics, who nonetheless met the criteria for vaginal delivery after professional assessment by obstetricians; and 3) neonate mothers who did not undergo painless delivery. Neonate mothers who had breech delivery and vaginal birth after cesarean section were excluded. Neonates who had the following abnormal conditions were excluded: stillbirth, fetal death *in utero*, and neonatal congenital malformation.

In total, 10,550 neonates were included in the study. Clinical records and data pertaining to the stages of labor and neonatal condition at birth were collected.

### Variables

The maternal clinical characteristics recorded included age at delivery, highest level of education attained, body mass index (BMI) during the week before delivery, gestational age, parity, pathological obstetrics, hemoglobin (Hb) level during the week before delivery, first stage of labor, second stage of labor, and forceps delivery or vacuum extraction. The neonatal clinical characteristics recorded included weight, sex, and presence of meconium-stained amniotic fluid (MSAF).

In this study, gestational age (recorded in completed weeks) was calculated from the last menstrual period (LMP) to the date of delivery. When the mothers forgot their LMP or had irregular menstruation, information about the LMP was obtained from the first B-scan ultrasound. Pathological obstetrics included pregnancy-specific disorders and pregnancy complicated by a medical disease. Pregnancy-specific disorders included term premature rupture of membranes, gestational diabetes mellitus, hypertensive disorders complicating pregnancy, marginal placenta previa, polyhydramnios, chorioamnionitis, and oligohydramnios. In this study population, medical and/or surgical diseases complicating pregnancy included chronic asymptomatic hepatitis B virus carrier status; mildly abnormal function of the liver, kidney, heart, or thyroid gland; diabetes; thrombocytopenia; and hysteromyoma. However, after professional assessment by obstetricians, all patients met the criteria for vaginal delivery.

### Apgar scoring system

The overall Apgar score is the sum of five components, including heart rate, respiratory effort, muscle tone, reflex irritability, and skin color. A score of 7 to 10 is defined as reassuring and a score of 0 to 6 is defined as abnormal. After vaginal birth, an obstetrician and a midwife evaluate the neonates and assign Apgar scores.

### Assessment of risk factors for low 1-min Apgar scores

Patients were categorized as having normal (≥7) or low (<7) 1-min Apgar scores. Maternal and neonatal clinical factors were compared between the normal and low 1-min Apgar score groups. The association between 1-min Apgar score and maternal and neonatal clinical factors was examined.

### Missing data

This study included mothers and newborns; therefore, data that were missing from a mother's records were completed on the basis of information obtained from the newborn's record and vice versa. Any other missing data were obtained through the use of mean imputation.

### Statistical analysis

The two-tailed Student's *t*-test, chi-squared test, and Fisher's exact test were used to compare dichotomous variables between groups. Binary logistic regression analysis was used for univariate analysis to determine the association between variables and low 1-min Apgar scores. Backward stepwise logistic regression analysis was used for multivariate analysis to identify the independent risk factors for low 1-min Apgar scores. All variables were entered into the multivariate logistic regression model in order to identify as many independent risk factors as possible. The analysis performed before stepwise regression showed that there was no collinearity among the variables. All statistical analyses were performed using SPSS ver. 24.0 (IBM Inc, USA). P-values <0.05 were considered statistically significant.

## Results

### Patient characteristics

**Figure 1:**
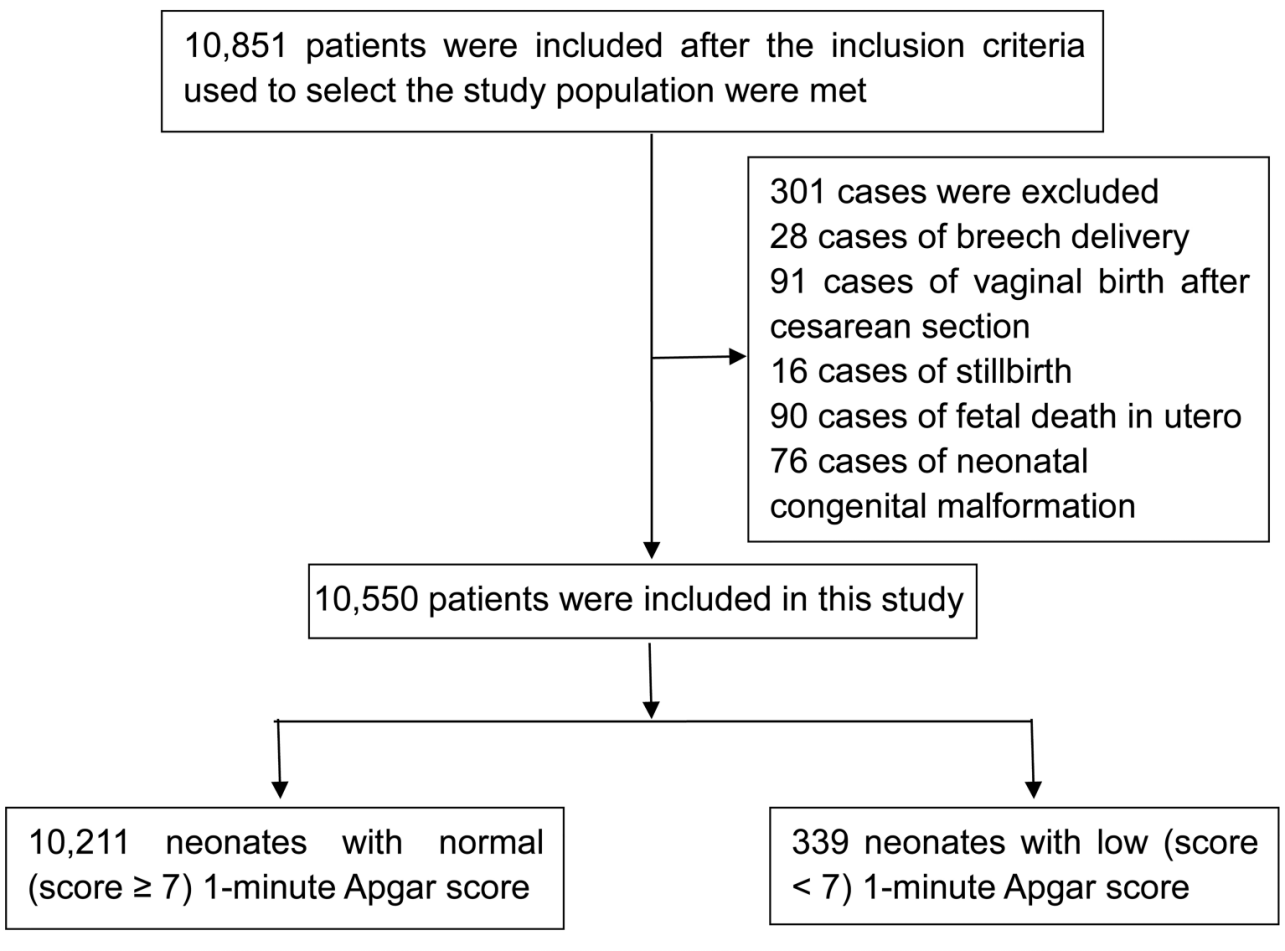
Flow-chart for patient selection process.

A flow-chart for the neonatal selection process is presented in Figure 1. Ultimately, 10,550 neonates were included in this study. The clinical characteristics of neonates and their mothers are summarized in Table 1. Among the neonates, 10,211 (96.8%) had 1-min Apgar scores ≥7, and 339 (3.2%) had 1-min Apgar scores <7. A total of 1535 (14.5%) neonates were admitted to the neonatology department for further observation or treatment and all were healthy at the time of discharge.

**Table 1:** Maternal and neonatal clinical characteristics of single-gestation infants born through vaginal delivery from 37 weeks 0 days to 41 weeks 6 days.

Characteristics	
Maternal characteristics	
Age at delivery, mean (range), years	29.5 (16–46)
Highest level of education attained, n (%)	
Junior high school and below	3546 (33.6)
Senior high school	1917 (18.2)
College and higher	5087 (48.2)
BMI during the week before delivery, mean (SD), kg/m^2^	26.3 (3.0)
Gestational age, mean (SD), weeks	39.5 (1.1)
Parity, n (%)	
1	4938 (46.8)
≥2	5612 (53.2)
Pathological obstetrics, n (%)	
No	5243 (49.7)
Yes	5307 (50.3)
Hb level during the week before delivery, n (%), g/L	
<110	3058 (29.0)
≥110	7492 (71.0)
First stage of labor, mean (SD), h	6.2 (3.5)
Second stage of labor, mean (SD), min	28.3 (28.2)
Forceps delivery or vacuum extraction, n (%)	
No	10,136 (96.1)
Yes	414 (3.9)
Neonatal characteristics	
Weight, mean (SD), g	3245.7 (364.0)
Sex, n (%)	
Male	5747 (54.5)
Female	4803 (45.5)
MSAF, n (%)	
No	7960 (75.5)
Yes	2590 (24.5)
Admission to the neonatology department	1535 (14.5)
Mortality	0 (0)

BMI: body mass index; Hb: hemoglobin; MSAF: meconium-stained amniotic fluid.

### Comparison of maternal and neonatal clinical characteristics

Maternal and neonatal clinical characteristics were compared between the normal and low 1-min Apgar score groups (Table 2). There was no statistically significant difference between the normal and low 1-min Apgar score groups in terms of the age at delivery (P=0.248), highest level of education attained (P=0.287), and neonatal sex (P=0.172). Conversely, there were statistically significant differences in BMI during the week before delivery (P=0.002), gestational age (P<0.001), parity (P<0.001), pathological obstetrics (P<0.001), Hb level during the week before delivery (P=0.042), duration of the first stage of labor (P<0.001), duration of the second stage of labor (P<0.001), forceps delivery or vacuum extraction (P<0.001), neonatal weight (P<0.001), MSAF (P<0.001), and admission to the neonatology department (P<0.001). The rate of admission to the neonatology department was higher in the low 1-min Apgar score group than in the normal 1-min Apgar score group (94.7% *vs* 11.9%) (OR, 132.16; 95%CI, 81.90-213.27; P<0.001).

**Table 2:** Comparison of maternal and neonatal clinical characteristics in infants with normal 1-min Apgar scores (≥7) *vs* low 1-min Apgar scores (<7).

Characteristic	1-min Apgar scores		P value
	Score ≥7 (n=10,211)	Score <7 (n=339)	
Maternal characteristics			
Age at delivery, mean (SD), years	29.5 (4.7)	29.2 (4.5)	0.248
Highest level of education attained, n (%)			0.287
Junior high school and below	3420 (33.5)	126 (37.2)	
College and higher	4937 (48.3)	150 (44.2)	
BMI during the week before delivery, n (%), kg/m^2^			0.002
18.5-23.9	1609 (20.9)	27 (11.7)	
24-27.9	4131 (53.6)	133 (57.6)	
≥28	1962 (25.5)	71 (30.7)	
Gestational age, n (%), weeks			<0.001
37 0/7-38 6/7	3078 (30.1)	62 (18.3)	
39 0/7-40 6/7	6386 (62.5)	232 (68.4)	
41 0/7-41 6/7	747 (7.3)	45 (13.3)	
Parity, n (%)			<0.001
1	4732 (46.3)	206 (60.8)	
≥2	5479 (53.7)	133 (39.2)	
Pathological obstetrics, n (%)			<0.001
No	5154 (50.5)	89 (26.3)	
Yes	5057 (49.5)	250 (73.7)	
Hb level during the week before delivery, n (%), g/L			0.042
<110	2943 (28.8)	115 (33.9)	
≥110	7268 (71.2)	224 (66.1)	
First stage of labor, n (%), min			<0.001
<12	9572 (93.7)	294 (86.7)	
12-20	603 (5.9)	36 (10.6)	
>20	36 (0.4)	9 (2.7)	
Second stage of labor, n (%), min			<0.001
<60	9150 (89.6)	213 (62.8)	
60-120	899 (8.8)	72 (21.2)	
>120	162 (1.6)	54 (15.9)	
Forceps delivery or vacuum extraction, n (%)			<0.001
No	9896 (96.9)	240 (70.8)	
Yes	315 (3.1)	99 (29.2)	
Neonatal characteristics			
Weight, n (%), g			<0.001
<3250	5073 (49.7)	177 (52.2)	
3250-4000	4859 (47.6)	135 (39.8)	
≥4000	279 (2.7)	27 (8.0)	
Sex, n (%)			0.172
Male	5550 (54.4)	197 (58.1)	
Female	4661 (45.5)	142 (41.9)	
MSAF, n (%)			<0.001
No	7774 (76.1)	186 (54.9)	
Yes	2437 (23.9)	153 (45.1)	
Admission to the neonatology department			<0.001
No	8997 (88.1)	18 (5.3)	
Yes	1214 (11.9)	321 (94.7)	

BMI: body mass index; Hb: hemoglobin; MSAF: meconium-stained amniotic fluid.

### Risk factors for low 1-min Apgar scores

The risk factors for low 1-min Apgar scores, as determined by univariate and multivariate analyses of maternal and neonatal clinical data, are listed in Table 3. The results of multivariate analysis revealed that highest level of education attained, BMI during the week before delivery, gestational age, pathological obstetrics, duration of the second stage of labor, forceps delivery or vacuum extraction, neonatal weight, neonatal sex, and presence of MSAF were independent risk factors for low 1-min Apgar scores. The subgroup analysis of primiparous and multiparous women is shown in Table 4. Maternal age, BMI, and gestational age were found to be independent risk factors for low 1-min Apgar scores in multiparous women, compared with primiparous women.

**Table 3:** Univariate and multivariate analyses of risk factors for low 1-min Apgar scores (<7) in single-gestation neonates born vaginally from 37 weeks 0 days to 41 weeks 6 days.

Risk factor	Univariate analysis		Multivariate analysis	
	OR (95% CI)	P	OR (95% CI)	P
Maternal characteristics				
Age at delivery ≥35 years	0.973 (0.724-1.307)	0.853		
Highest level of education attained				
Junior high school and below	1		1	
Senior high school	0.922 (0.678-1.255)	0.607	0.798 (0.574-1.110)	0.180
College and higher	0.825 (0.648-1.049)	0.117	0.614 (0.469-0.804)	<0.001
BMI during the week before delivery, kg/m^2^				
18.5-23.9	1		1	
24-27.9	2.166 (1.450-3.236)	<0.001	2.146 (1.411-3.263)	<0.001
≥28	2.147 (1.371-3.360)	0.001	2.620 (1.637-4.195)	<0.001
Gestational age, weeks				
37 0/7-38 6/7	1		1	
39 0/7-40 6/7	1.804 (1.358-2.395)	<0.001	1.693 (1.248-2.297)	=0.001
41 0/7-41 6/7	2.991 (2.021-4.426)	<0.001		=0.001
Parity				
1	1			
≥2	0.558 (0.447-0.696)	<0.001		
Pathological obstetrics	2.863 (2.241-3.658)	<0.001	2.081 (1.604-2.700)	<0.001
Hb level during the week before delivery, g/L				
<110	1		1	
≥110	0.789 (0.627-0.992)	0.042	0.799 (0.623-1.025)	0.078
First stage of labor, h				
<12	1			
12-20	0.944 (1.362-2.774)	<0.001		
>20	8.139 (3.885-17.053)	<0.001		
Second stage of labor, min				
<60	1		1	
60-120	3.440 (2.611-4.533)	<0.001	3.082 (2.268-4.188)	<0.001
>120	14.319 (10.227-20.050)	<0.001	5.017 (3.332-7.554)	<0.001
Forceps delivery or vacuum extraction	12.959 (9.996-16.800)	<0.001	7.030 (5.074-9.738)	<0.001
Neonatal characteristics				
Weight, g				
<3250	1		1	
3250-4000	0.796 (0.634-1.000)	0.050	0.452 (0.334-0.611)	<0.001
≥4000	2.774 (1.818-4.232)	<0.001	1.497 (0.903-2.484)	0.118
Sex				
Male	1		1	
Female	0.858 (0.689-1.069)	0.172	0.618 (0.485-0.787)	<0.001
MSAF	2.624 (2.109-3.265)	<0.001	1.882 (1.481-2.392)	<0.001

BMI: body mass index; Hb: hemoglobin; MSAF: meconium-stained amniotic fluid; OR: odds ratio; CI: confidence interval. The Hosmer and Lemeshow test and the chi-squared test=12.40 (P=0.13, R-square=0.293) for binary multivariate logistic regression were used.

**Table 4:** Multivariate analyses of risk factors for low 1-min Apgar scores (<7) in the primiparous and multiparous subgroups.

Risk factor	Multivariate analysis (primiparas)		Multivariate analysis (multiparas)	
	OR (95% CI)	P	OR (95% CI)	P
Maternal characteristics				
Age at delivery ≥35 years	2.396 (1.561-3.676)	<0.001		
Highest level of education attained				
Junior high school and below	1		1	
Senior high school	0.958 (0.604-1.521)	0.856	1.238 (0.870-1.762)	0.235
College and higher	0.684 (0.464-1.009)	0.055	0.383 (0.258-0.570)	<0.001
BMI during the week before delivery, kg/m^2^				
18.5-23.9	1			
24-27.9	1.444 (0.787-2.652)	0.236		
≥28	2.238 (1.071-4.679)	0.032		
Gestational age, weeks				
37 0/7-38 6/7	1			
39 0/7-40 6/7	2.686(1.771-3.854)	<0.001		
41 0/7-41 6/7	3.092 (2.191-4.364)	<0.001		
Pathological obstetrics	2.650 (1.851-3.793)	<0.001	1.534 (1.020-2.308)	0.040
Hb level during the week before delivery, g/L				
<110				
≥110				
First stage of labor, h				
<12				
12-20				
>20				
Second stage of labor, min				
<60	1			
60-120	1.575 (0.596-4.158)	0.359	2.489 (2.012-13.079)	<0.001
>120	4.251 (2.083-8.677)	<0.001	5.922 (4.055-8.931)	<0.001
Forceps delivery or vacuum extraction	6.163 (4.169-9.111)	<0.001	15.139 (5.837-39.265)	<0.001
Neonatal characteristics				
Weight, g				
<3250	1		1	
3250-4000	0.501 (0.303-0.827)	0.007	0.203 (0.159-0.378)	<0.001
≥4000	NA		1.422 (0.754-2.682)	0.277
Sex				
Male	1		1	
Female	0.710 (0.520-0.970)	0.031	0.335 (0.216-0.520)	<0.001
MSAF	1.418 (1.028-1.955)	0.033	2.485 (1.687-3.661)	<0.001

BMI: body mass index; Hb: hemoglobin; MSAF: meconium-stained amniotic fluid; OR: odds ratio; CI: confidence interval.

## Discussion

Studies on the Apgar score have reported that it is useful for conveying information about the newborn’s overall status and response to resuscitation efforts. Most previous studies focused on identifying the relationship between Apgar scores and neonatal outcomes. However, the risk factors that contribute to low 1-min Apgar scores have rarely been investigated. Traditionally, a 1-min Apgar score of 0 to 3 has not been assumed to predict the long-term outcome of an individual neonate. A 5-min Apgar score of 0 to 3 correlates with long-term neonatal mortality in large populations (3,5). However, recently, a population-based cohort study of term infants in Sweden demonstrated that 1-min Apgar scores in the range of 7–9 are strongly associated with neonatal mortality and morbidity, compared with the 1-min Apgar score of 10 (6). In addition, the 1-min Apgar score reflects neonatal status at birth, and our study showed that a low 1-min Apgar score was associated with a higher rate of admission to the neonatology department. Therefore, the risk factors that contribute to low 1-min Apgar scores are significant and deserve investigation.

Several previous studies have reported that maternal education is an important socioeconomic predictor of adverse birth outcomes, both independently of as well as in combination with other maternal socioeconomic indicators (7,8). In this study, univariate analysis showed that maternal education was not associated with low 1-min Apgar scores. However, multivariate analysis showed that maternal education was an independent risk factor for low 1-min Apgar scores. Women with higher levels of education may have better compliance to prenatal exams. Numerous studies have demonstrated that maternal overweight or obesity may increase the risks of stillbirth and infant mortality, independently of genetic and early environmental risk factors that are shared within families (9,10). Maternal obesity and hyperglycemia are well-known independent risk factors for fat deposition in newborns; however, excess adiposity at birth may be present even in the absence of these risk factors (11). However, the above-mentioned studies did not investigate the relationship between maternal weight and Apgar scores. This study’s subgroup analysis demonstrated that multiparous maternal overweight, combined with the above-mentioned adverse outcomes, contributed to low 1-min Apgar scores. Hence, weight control in pregnant women especially multipara can improve the status of neonates at birth.

Pathological obstetrics is common during pregnancy, and our data showed that morbidity was associated with pathological obstetrics in 50.3% of cases. In order to extend the clinical application of these findings, our study included not only normal pregnant women but also women with pathological pregnancies. Because pathological obstetrics are common and this study population was relatively limited compared to that of other studies, which included more than 100,000 participants, we have merged the factors related to pathological obstetrics. Many previous studies have reported that medical diseases, including cardiovascular disease, thyroid disease, diabetes mellitus, kidney disease, and liver disease, are associated with adverse pregnancy outcomes (12–16). Our study demonstrated that pathological obstetrics also contributed to low 1-min Apgar scores.

The second stage of labor begins with complete cervical dilation and ends with fetal birth. The median duration of the second stage of labor varies broadly; the reported values are approximately 50 min for nulliparas and approximately 20 min for multiparas (17). The results of our study are in accordance with this finding. The American College of Obstetricians and Gynecologists and the Society for Maternal-Fetal Medicine have provided recommendations for the second stage of labor. In cases that do not require epidural analgesia and are not complicated by fetal malposition, physicians should allow at least 2 h of pushing in multiparous women and at least 3 h of pushing in nulliparous women (18). Several retrospective studies of nulliparous women did not find any association between a neonatal 5-min Apgar score <4 and the duration of the second stage of labor (19,20). However, many studies of multiparous women demonstrated that the risks of a 5-min Apgar score <7 and admission to the neonatal intensive care unit were significantly increased when the second stage of labor exceeded 2 h (21,22). Moreover, prolongation of the second stage of labor is associated with an increased risk of morbidity and a decreased probability of spontaneous vaginal delivery (19,21,22).

This study did not include the data of cases that underwent cesarean delivery, as it aimed to demonstrate the association between stages of labor and 1-min Apgar scores. We demonstrated that a longer duration of the second stage of labor is associated with an increased risk of low 1-min Apgar scores. Compared to a second stage of labor that lasts <60 min, a second stage of labor that lasts 60-120 or >120 min is associated with increases of approximately three-fold and five-fold, respectively, in the risk of low 1-min Apgar scores. The reason for obtaining this result may be that this study included multiparous women who tend to have a shorter second stage of labor (17). In the subgroup analysis, primiparous women who had a second stage of labor lasting >120 min and multiparous women who had a second stage of labor lasting >60 min were found to have an increased risk for low 1-min Apgar scores.

Gestational age is one of the most important factors affecting Apgar score. The healthy preterm newborn with no evidence of asphyxia may receive a low score because of immaturity alone (23). Therefore, our study did not include premature infants. Previous studies demonstrated that neonatal mortality is lowest among uncomplicated pregnancies delivered between 39 weeks 0 days and 41weeks 0 days (3,24). Interestingly, data from the subgroup analysis of multiparas revealed that birth from 37 weeks 0 days to 38 weeks 6 days, compared with birth from 39 weeks 0 days to 41 weeks 6 days, protects against a low 1-min Apgar score. This finding reflects the subjective components included in the Apgar scoring system, as reported in a previous study that documented the limited external validity of cross-national comparisons (25). On the other hand, numerous factors can influence the Apgar score. The Apgar score is conveniently used to assess neonatal status. However, it has been inappropriately used to diagnose asphyxia and to predict adverse neurologic outcomes. Hence, a low 1-min Apgar score may reflect the status at birth but not the risk of long-term mortality. However, this study aimed to identify independent risk factors for low 1-min Apgar scores and to improve the status of newborns. This study was not designed to investigate the association of Apgar score with long-term outcomes.

A previous study demonstrated that low 1-min Apgar scores (<3) were significantly associated with birth weight in premature infants (23). As mentioned above, Apgar scores may be influenced by a variety of factors, such as prematurity. Our study showed that this correlation existed not only in premature infants but also in term infants. In addition, our study showed that neonatal birth weight between 3250–4000 g would be optimal and that female neonates were at decreased risk of low (score <7) 1-min Apgar scores. Similarly, a large population-based study found that male sex was independently associated with neonatal death (26). This could be because female neonates have better hypoxia tolerance than do male neonates.

Evidence shows that the incidence of MSAF increases along with gestational age. We found that 24.5% of patients had MSAF; this finding was consistent with previous studies in which 7–22% of term pregnancies were complicated by MSAF (27,28). MSAF was associated with poor perinatal outcomes such as meconium aspiration syndrome. A previous study demonstrated that MSAF is associated with low Apgar scores (29). However, the study did not present Apgar scores at any particular time-point. Our study showed that the presence of MSAF increased the risk of low 1-min Apgar scores. Although we did not analyze the rate of subsequent development of meconium aspiration syndrome, the presence of MSAF in combination with low 1-min Apgar scores is likely to greatly increase the risk of meconium aspiration syndrome.

The above-mentioned independent risk factors for low 1-min Apgar scores, including educational background, gestational age, pathological obstetrics, forceps delivery or vacuum extraction, neonatal sex, and MSAF, generally cannot be assessed when mothers undergo their first prenatal examination. However, BMI, longer duration of the second stage of labor, and neonatal weight can actually be controlled. Weight control and balanced nutrition during pregnancy and strict labor management may improve neonatal outcomes at birth.

This study may have an inherent selection bias due to its retrospective nature. There is also the possibility of inter-observer variation. In addition, many factors can influence neonatal outcomes at birth. However, we could not take all factors into account in this study, such as the indicator of infection. This is because parents of few neonates agree to further examination when there is only a suspicion of infection. Therefore, although we tried our best to include all possible clinical risk factors, some risk factors could not be included in this study. Furthermore, a 1-min Apgar score <3 is usually considered as a low score. However, only a limited number of neonates with 1-min Apgar scores <3 were included in this study. In addition, a large population-based cohort study found 1-min Apgar scores in the range of 7–9 were meaningful indicators of neonatal mortality and morbidity (6). Thus, scores <7 were considered to be low 1-min Apgar scores in this study.

In conclusion, early detection of risk factors and timely intervention to address these risk factors may improve neonatal outcomes at birth and reduce the rate of admission to the neonatology department.
